# Disparities in cancer prevalence, incidence, and mortality for incarcerated and formerly incarcerated patients: A scoping review

**DOI:** 10.1002/cam4.4251

**Published:** 2021-09-03

**Authors:** Christopher R. Manz, Varshini S. Odayar, Deborah Schrag

**Affiliations:** ^1^ Division of Population Sciences Department of Medical Oncology Dana‐Farber Cancer Institute Boston MA USA; ^2^ Harvard Medical School Boston MA USA; ^3^ Harvard University Boston MA USA

**Keywords:** cancer, criminal justice, healthcare disparities, incarceration, jail, prison, scoping review

## Abstract

**Background:**

Racial and ethnic minority status, structural racism, low educational attainment, and poverty are consistently associated with cancer disparities and with higher rates of incarceration. The objective of this scoping review is to conduct a qualitative synthesis of the literature on cancer prevalence, incidence, mortality, and disparities in these outcomes for incarcerated and formerly incarcerated patients, as this literature is fragmented and heterogenous.

**Methods:**

This scoping review included Bureau of Justice Statistics reports and searched PubMed in May 2021 for all English language studies published between 1990 and 30 April 2021, that reported on cancer prevalence, incidence, or mortality for incarcerated or formerly incarcerated individuals in the United States.

**Results:**

Twenty studies were selected. Data on cancer prevalence and incidence were scarce but suggested that incarcerated and formerly incarcerated patients have a similar overall risk of cancer diagnosis as the general population, but elevated risk of certain cancers such as cervical, lung, colorectal, and hepatocellular carcinoma for which effective prevention and screening interventions exist. Cancer mortality data in state and local jails as well as prisons were robust and suggests that both incarcerated and formerly incarcerated patients have higher cancer mortality than the general population.

**Conclusions:**

Incarcerated and formerly incarcerated patients likely have a higher risk of dying from cancer than the general population, but important gaps in our knowledge about the extent and drivers of disparities for this population remain. Additional research is needed to guide interventions to reduce cancer disparities for patients experiencing incarceration.

## Graphical abstract

This scoping review finds that individuals who are currently or formerly incarcerated in prison have higher cancer mortality than the general population. However, important knowledge gaps remain that must be addressed to inform targeted interventions to reduce cancer disparities in prisons and in the community.

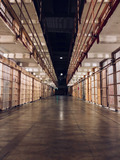

## 1 INTRODUCTION

Despite decades of progress in the prevention and treatment of cancer in the United States, disparities in cancer incidence and survival are significant.^1^ Factors such as racial/ethnic minority status, structural racism, low educational attainment, and poverty have been consistently associated with a higher incidence of cancer, late diagnosis, and worse survival.^1^ Nearly all of these disadvantaging factors converge upon one particularly vulnerable population: incarcerated and formerly incarcerated individuals. For instance, Black men are incarcerated at nearly six times the rate for White men,^2^ and Black men have higher cancer mortality than White men for lung cancer (52 vs. 45 per 100,000 of Black and White men, respectively), colorectal cancer (22 vs. 16 per 100,000), and liver cancer (13 vs. 9 per 100,000), among other preventable cancer types.^3^

The United States has a large and aging incarcerated population that faces an enormous rise in cancer risk. Nearly 2.1 million individuals were incarcerated in local, state, and federal jails and prisons in 2018.^4^ The population of individuals incarcerated in US prisons aged 55 years or older (i.e., those at highest risk for cancer) grew 14‐fold from 8853 in 1981 to 124,900 in 2010, and is expected to reach 400,000 by 2030.^5^ Unsurprisingly, cancer deaths are rising in these prisons.

There are no comprehensive studies describing the relationship between incarceration and cancer incidence and mortality. Though often outsourced to private health companies, federal, state, and local governments are responsible for healthcare for individuals incarcerated in the nation’s jails and prisons. As a result, healthcare data for incarcerated patients are fragmented and the population is absent from disparities analyses based upon Medicare, Medicaid, or commercial claims databases. Cancer diagnoses are reported to state cancer registries and thus included in analyses using these databases, but in the absence of time‐consuming linkage to incarceration records, disparities related to incarceration status are largely invisible in these studies. A 2004 national survey found that incarcerated individuals had 22% higher odds of a cancer diagnosis compared to the general population,^6^ while cancer caused 27% of deaths in state prisons between 2001 and 2016, with mortality rates rising 59% over that period (from 58 to 92 per 100,000 individuals incarcerated in state prisons).^7^ However, data on cancer incidence and mortality rates, particularly compared to the general population, are limited in scope, geography, and applicability.

Cancer control efforts to address disparities for vulnerable populations experiencing incarceration are stymied by this complex literature and the lack of clear data upon which to base interventions to improve cancer care. A scoping review is a methodology to systematically evaluate knowledge gaps in the literature^8^; to inform future cancer control efforts, this study conducts a scoping review of the existing literature on cancer prevalence, incidence, mortality, and racial and ethnic disparities in these measures for individuals who were incarcerated or formerly incarcerated at the time of their cancer diagnosis.

## 2 METHODS

### 2.1 Study design and data sources

We conducted a scoping literature review using PubMed, an online biomedical literature database consisting of over 32 million citations from MEDLINE, life science journals, and online books. This study was conducted in accordance with the Preferred Reporting Items for Systematic Reviews and Meta‐Analysis––Scoping Review (PRISMA‐ScR) guidelines (Table S1). As a review of published studies, this study is exempt from review and was not submitted for approval from an Institutional Review Board.

### 2.2 Article selection

In May 2021, we searched PubMed for all English language articles published between 1 January 1990 and 30 April 2021, and included the search terms: cancer AND (prevalence OR incidence OR mortality) AND (jail OR prison OR incarcerat* OR “criminal justice” NOT hernia). PubMed was last searched on 18 June 2021 to capture articles that were published by 30 April 2021 but had not been indexed in PubMed at the time of the initial search in May 2021. Articles on incarcerated hernias were excluded. Abstracts were independently reviewed by two investigators (CM and VO) for initial screening, as were full text articles reviewed after the initial screening. Articles were excluded if they did not report data on the study outcomes of cancer prevalence, incidence, or mortality for incarcerated or formerly incarcerated patients, or if the study population was not based in the United States. Inconsistent scoring after independent selection was resolved by discussion and consensus of study authors.

In addition to articles included through the PubMed search, two study investigators (CM and VO) reviewed references in included articles for additional studies that met inclusion criteria. Finally, study investigators reviewed and included the most recent reports from the US Bureau of Justice Statistics, which publishes annual reports describing deaths that occur in US jails and prisons. This review was not registered; the review protocol, templates used for data collection, and original data collection spreadsheets are available upon request.

### 2.3 Data synthesis and analysis

For each included article, we abstracted whether the study involved incarcerated patients, formerly incarcerated patients, or both; whether the study population was in jail or prison; whether the correctional facilities were local, state, or federal; study population characteristics including number of patients, sex, years of inclusion, and location; source of data; and primary findings regarding (1) cancer prevalence, incidence, or mortality, as absolute numbers or rates and subdivided by cancer type when available, for incarcerated or formerly incarcerated patients, (2) comparison of findings to non‐incarcerated populations, if reported; and (3) reported racial or ethnic disparities in any of the findings. Two investigators (CM and VO) conducted the abstraction independently, results were merged by consensus, and reviewed by the third investigator (DS). The qualitative synthesis of major findings regarding cancer prevalence, incidence, and mortality in the included studies was summarized in a table.

## 3 RESULTS

**FIGURE 1:**
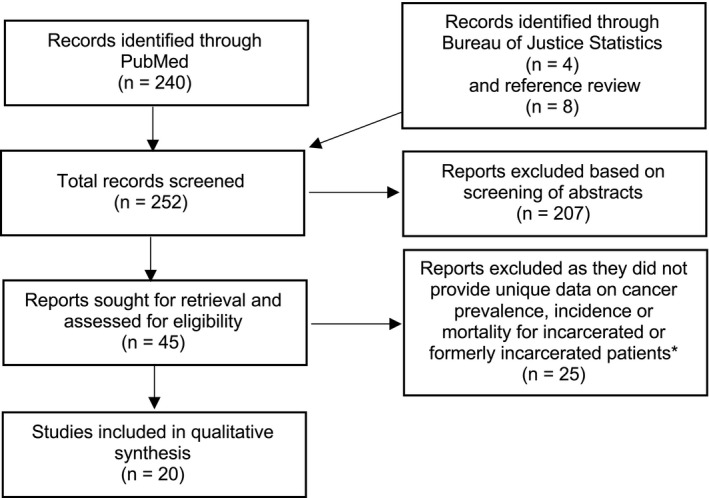
PRISMA flow diagram

A total of 252 studies were screened and assessed for eligibility, and 20 were included in this review (Figure 1). Of note, one study^9^ was excluded that contained a subset of data reported in another, parent publication that was included in this review.^10^ Three other studies fit this criteria but were nonetheless included in this review because the studies reported additional data or analyses that were not included in the parent papers (see Table 1).^11^, ^12^, ^13^ Two additional studies were excluded as they provided data only on cancer deaths in a hospital^14^ and cancer prevalence in a small study of individuals in prison who smoke^15^ as these studies do not reflect cancer prevalence, incidence, or mortality in jail or prison populations as a whole.

**TABLE 1:** Characteristics of included studies

	*n* (%)
Total included studies	20 (100)
Correctional facilities	
Prison	18 (90)
Jail	4 (20)
Government jurisdiction	
Local	4 (20)
State	18 (90)
Federal	2 (10)
Study population	
Incarcerated	16 (80)
Prevalence	7 (35)
Incidence	1 (5)
Mortality	11 (55)
Formerly incarcerated	7 (35)
Prevalence	1 (5)
Incidence	1 (5)
Mortality	6 (30)
Data from 2011 or later	5 (25)

Subcategories may sum to greater than 20 as a single study may contain multiple subcategories (e.g., jail and prison populations).

A summary of the characteristics of included studies is presented in Table 1 and a summary of study design and findings for the included studies are presented in Table 2. Of the 20 studies included in this review, 16 studies included prisons, two studies included jails, and two studies included both prisons and jails. Two, 18, and four studies examined federal, state, and local correctional facilities, respectively (sum is greater than 20 as some studies included multiple levels of government). Only five of the 20 studies used data that was less than 10 years old as of 2021.^7^, ^16^, ^17^, ^18^, ^19^ Except in studies sharing datasets (e.g., Spaulding et al^10^ and Zlotorynzska et al^12^), studies used incompatible methodologies, data sources, time periods, and patient populations that prevent direct quantitative comparisons of study findings. A qualitative review of major findings on cancer prevalence, incidence, mortality, and racial and ethnic disparities in those measures follows.

**TABLE 2:** Summary of included studies’ findings

Study	Prison or jail	Local, state, or federal	Prevalence, incidence, or mortality	Study population and data source	Findings
Studies involving incarcerated patients					
Bai et al. 2015^16^	Prison	New York	Prevalence	759 individuals (51% men) aged ≥16 years newly incarcerated in 2009–2011 at two maximum‐security prisons; medical records and patient interviews	Overall cancer prevalence: 1.7% Men: 0.8% Women: 2.7%
Baillargeon et al. 2004^20^	Prison	Texas	Prevalence	336,668 men and women (89% male) aged ≥18 years incarcerated in 1999–2001 in the Texas Department of Criminal Justice; incarceration electronic medical records	Prevalence of eight selected cancers per 100,000 incarcerated patients: Cervical (58), lung (27), colorectal (23), non‐Hodgkin's lymphoma (21), Hodgkin's lymphoma (21), leukemia (13), Kaposi sarcoma (4), and anal cancer (2)
Baillargeon et al. 2009^21^	Prison	Texas	Prevalence and Mortality	325,477 men aged ≥18 years incarcerated in 2003–2006 in the Texas Department of Criminal Justice; incarceration electronic medical records	*All rates per 100,000 of the respective populations* Hepatocellular carcinoma prevalence: 54 (vs. 7 in SEER) Hepatocellular carcinoma mortality rate: 33 (vs. 7 in SEER) Prevalence and mortality were higher among Hispanics (prevalence: 72; mortality 48) than African Americans (46; 28) or non‐Hispanic Caucasian individuals (48; 26). Hispanic individuals had a higher risk (OR: 2.9) and mortality (3.5) compared to non‐Hispanic individuals.
Binswanger et al. 2009^6^	Jail and Prison	National survey of local, state and federal facilities	Prevalence	Jail: 6157 (88% male) Prison: 13,931 (93% male) General Population: 76,597 (49% male); ages 18–65 years; 2002 Survey of Inmates in Local Jails, 2004 Survey of Inmates in State and Federal Correctional Facilities, 2002–4 National Health Interview Survey	All‐cancer and cervical cancer prevalence for age 50–65: ‐ General population: 9.1%, 1.1% ‐ Jail: 8.8%, 4.9% ‐ Prison: 9.0%, 5.4% In adjusted comparison to the general population: ‐ Odds of a cancer diagnosis were similar for jailed individuals (OR 1.19, not significant) but higher for imprisoned individuals (OR 1.22, significant) ‐ Odds of a cervical cancer diagnosis were higher for both jailed (OR 4.2) and imprisoned (OR 4.8) individuals.
Binswanger et al. 2010^22^	Jail	National survey of local jails	Prevalence	6982 persons aged ≥13 years incarcerated in local jails nationally (88% male); 2002 Survey of Inmates in Local Jails	Eight percent of women have reported having a history of cancer compared to 1% of men. Women had elevated odds of cancer compared to men (OR 7.7) that was no longer significant when gender‐specific cancers were removed (OR 1.5).
Carson et al. 2020^7^	Prison	State (Federal data did not include cancer)	Mortality	40,674 deaths during incarceration (96% male) aged ≥25 years in US state and federal prisons reported by the 2000–2016 Mortality in Correctional Institutions data collection from the Bureau of Justice Statistics	Cancer was the leading cause of death in 2016 and accounted for 16,777 deaths (27% of total) between 2000 and 2016. This increased from 24% of deaths in 2001 to 30% in 2016, with mortality rate rising from 58 to 92 per 100,000 individuals in state prisons. In 2016, cancer mortality rates per 100,000 varied by: ‐ Sex: men versus women (91 vs. 53) ‐ Race: White versus Black versus Hispanic (154 vs. 89 vs. 38) ‐ State: ranging from 20 (Connecticut) to 160 (Louisiana)
Carson 2021^17^	Jail	National survey of local jails	Mortality	12,009 deaths during incarceration (87% male) of all ages in US local jails reported by the 2000–2018 Mortality in Correctional Institutions data collection from the Bureau of Justice Statistics	Cancer accounted for 698 deaths (3.6% of total) from 2000 to 2018. Cancer mortality was: ‐ Five per 100,000 locally jailed individuals ‐ Five versus four per 100,000 for men versus women, respectively ‐ Seven, six, and three per 100,000 for White, Black, and Hispanic individuals, respectively Median time in jail to cancer death was 137 days. Sex, age‐, and race/ethnicity‐adjusted cancer mortality were lower in jailed individuals compared to the US population (4 vs. 59 per 100,000).
Harzke et al. 2009^25^	Prison	Texas	Mortality	4026 men aged 25–84 years who died while incarcerated in Texas state prisons between 1992 and 2003, emphasis on liver cancer; Texas Department of Criminal Justice records	Liver cancer accounted for 134 deaths (3% of total). Age‐ and race‐adjusted liver cancer death rates per 100,000 were ‐ 11.8 for the total population ‐ 17.6, 13.6, and 6.8 for Hispanic, White, and Black patients, respectively The standardized mortality ratio (SMR) increased from 7 per 100,000 between 1992 and 1994 to 15.6 between 2001 and 2003. In comparison, the SMR between 2001 and 2003 was 3.8 and 5.1 for age‐adjusted non‐incarcerated men in Texas and the United States, respectively (from CDC‐WONDER database).
Harzke et al. 2011^26^	Prison	Texas	Mortality	4026 men aged 25–84 years who died while incarcerated in Texas state prisons between 1992 and 2003; Texas Department of Criminal Justice records	Cancer accounted for 953 deaths (21% of total) and grew from 18% of deaths in 1992–1995 to 28% in 2000–2003, the leading cause of death during that period. Lung (10% of all deaths), liver (4%), and blood (3%) cancers were the most common cancers in 2000–2003. Cancer mortality in 2000–2003 per 100,000 incarcerated mem: ‐ Crude rate: 95 ‐ Age‐ and race‐adjusted to match the Texas male population: 207 Between 2000 and 2003, non‐Hispanic White and Black patients had higher cancer mortality rates than Hispanic patients (94 vs. 84 vs. 71 per 100,000 incarcerated men, respectively)
Mathew et al. 2005^23^	Prison	Texas	Prevalence and mortality	1807 persons (89% men) aged 15–87 years incarcerated in Texas state prisons and diagnosed with cancer in 1980–1999; Texas Department of Criminal Justice records and comparative US SEER population (date range not specified)	Cancer diagnoses increased 10‐fold from 1980 to 1999 (specific data not presented), tightly correlated with prison population growth (*r* ^2 ^= 0.9). Of 1807 diagnoses, there were 1048 deaths (58%). Top five cancer diagnoses for incarcerated individuals compared to a random SEER sample: Lung (25% vs. 14%), Non‐Hodgkin's lymphoma (8% vs. 3%), oral (8% vs. 3%), Colon (6% vs. not available), and prostate (6% vs. not available). Among women, cervical cancer was the most common (32% vs. 12%). Median overall survival was 21 months for incarcerated patients versus 54 months for age, gender, and race‐matched SEER‐Medicare patients
Mumola 2007^11^	Prison	All States	Mortality	12,129 deaths (96% men) aged ≥15 years reported in the Deaths in the Custody Reporting Program by all state prison systems, 2001–2004	*This study reports on a subset of data from Carson 2021. *Findings unique to this study are displayed*. Cancer accounted for 2820 deaths (23% of total). Lung cancer accounted for more deaths than the next six cancers combined. Top causes of cancer deaths were lung (910 deaths), liver (276), colon (171), pancreas (124), non‐Hodgkin's lymphoma (114), and prostate (92). Cancer mortality for individuals who had served <60, 60–119, and ≥120 months was 38, 70, and 151 per 100,000 prisoners. Cancer was present at time of admission to prison in 54%.
Rosen et al. 2011^27^	Prison	North Carolina	Mortality	105,237 men aged 20–79 incarcerated in state prison in 1994–2005; state prison and death records	Cancer caused 24% of prisoner deaths compared to 28% for the general population. ‐ Compared to non‐incarcerated White men, White incarcerated men had higher standardized mortality ratio (SMR) for all cancers (1.5), liver cancer (5.1), and lung cancer (2.1). ‐ Compared to non‐incarcerated Black men, Black incarcerated men had lower SMR for all cancers (0.7), but similar SMR for lung and liver cancers.
Rosen et al. 2012^24^	Prison	National survey of all states	Prevalence	8795 men aged ≥16 years incarcerated in state prisons who participated in the 2004 Survey of Inmates in State Correctional Facilities	White men reported having a higher age‐adjusted history of ever having cancer (1.3%) compared to black men (0.4%).
Studies that include mixed incarcerated and formerly incarcerated patients						
Binswanger et al. 2007^13^	Prison	Washington	Mortality	30,237 incarcerated individuals (87% male) aged ≥18 years released from Washington State Department of Corrections in 1999–2003; Washington State Department of Corrections, National Death Index and CDC‐WONDER (for comparison)	*This study reports on a subset of data from Binswanger 2013. *Findings unique to this study are displayed*. Crude mortality rates for incarcerated versus formerly incarcerated patients was 42 versus 68 per 100,000 person‐years.
Spaulding et al. 2015^10^	Prison	Georgia	Mortality	23,510 individuals (94% men) of all ages incarcerated on 30 June 1991 and observed through 2010; Georgia Department of Corrections and National Death Index	Cancer caused 143 (22%) of deaths inside of prison and 462 deaths (15%) of deaths) after release from prison. Cancer was the second leading cause of death in both time periods. Seventy‐eight percent of cancer deaths occurred after release from prison. Figures exclude 51 deaths from liver cancer and HIV‐associated cancers for which timing is not provided.
Zlotoryznska et al. 2016^12^	Prison	Georgia	Incidence, Mortality	22,354 individuals (94% men) aged ≥20 years incarcerated on 30 June 1991, still alive in 1998 and observed through 2010; Georgia Department of Corrections, Georgia Comprehensive Cancer Registry and National Death Index *Note*: *Patients could be incarcerated*, *in the general population or alternating between both populations during the observation period*.	*This study reports on a subset of data from Spaulding 2015. *Findings unique to this study are displayed*. Incidence: ‐ There were 847 cancers diagnosed, 49 in patients with HIV. Cancer incidence for those with versus without HIV was 561 versus 304 per 100,000 person‐years. ‐ Among HIV‐negative patients (96% of population): ‐ Leading diagnoses: lung, prostate, colorectal, kidney, and non‐Hodgkin lymphoma (75, 63, 31, 13, and 11 per 100,000, respectively). ‐ Thirty‐six percent presented with localized, 23% with regional, and 32% with distant cancer, with 9% unknown. ‐ All‐cancer incidence was lower than the Georgia general population (Standardized incidence ratio 0.9, 95% CI 0.8–0.9) Mortality: ‐ There were 481 cancer deaths, 12 in patients with HIV. The mortality rate was 181 per 100,000. ‐ Leading cancer deaths in non‐HIV patients were: lung, colorectal, liver, prostate, and stomach (74, 16, 11, 8, and 6 per 100,000, respectively). ‐ All‐cancer mortality was higher than the Georgia general population (Standardized mortality ratio 1.2, 95% CI 1.1–1.3)
Studies involving formerly incarcerated patients						
Binswanger et al. 2013^28^	Prison	Washington	Mortality	76,208 incarcerated individuals (84% men) aged 18–84 years released from Washington State prisons in 1999–2009; Washington State Department of Corrections, National Death Index and CDC‐WONDER	Cancer was the third leading cause of death, occurring in 71 per 100,000 person‐years. Lung and liver cancer deaths were the most common (26 and 10 per 100,000 person‐years). Formerly incarcerated individuals had a cancer standardized mortality ratio of 1.9 compared to the sex, age, and race‐matched US general population in CDC‐WONDER.
Jones et al. 2017^18^	Prison	North Carolina	Mortality	41,495 individuals (89% men) aged ≥20 years formerly incarcerated in North Carolina prisons released between 2008 and 2010; North Carolina Dept of Public Safety and Dept of Health and Human Services	By the end of 2012, cancer was the second leading cause of death. Released individuals had a cancer age‐adjusted standardized mortality ratio of 3.9 compared to the general population.
Puglisi et al. 2020^19^	Prison and Jail	Local, state, or federal	Prevalence	85,785 individuals aged ≥18 years responding to the 2008–2017 National Surveys on Drug Use and Health	15,563 individuals (71% men) with criminal justice involvement in the prior year had higher cancer prevalence compared to those without criminal justice involvement for: ‐ Lung cancer (4.6% vs. 2.4%) ‐ Cervical cancer (13.8% vs. 9.1%) ‐ Alcohol‐related cancers (3.5% vs. 1.7%) Cancer prevalence was similar for colon, breast, prostate, and smoking‐related cancers.
Rosen et al. 2008^29^	Prison	North Carolina	Mortality	15,673 men aged 20–69 years incarcerated in North Carolina prisons and released between 1980 and 2004 and died by the end of 2005; North Carolina Dept of Public Safety and state death records	A smaller proportion of formerly incarcerated patients died from cancer compared to the general population (14.7% vs. 26.3%). Compared to the general population, the age‐adjusted standardized mortality ratio for: ‐ White formerly incarcerated individuals was higher for all cancers (1.27), liver cancer (3.3), and lung cancer (1.7) ‐ Black formerly incarcerated individuals was lower for all cancers (0.74) and for lung cancer (0.84), but higher for liver cancer (1.7)

### 3.1 Studies involving incarcerated patients

Out of 16 included studies for incarcerated patients, including three studies with mixed populations of incarcerated and formerly incarcerated patients, seven reported on prevalence,^6^, ^16^, ^20^, ^21^, ^22^, ^23^, ^24^ one reported on incidence,^12^ and 11 reported on mortality.^7^, ^10^, ^11^, ^12^, ^13^, ^17^, ^21^, ^23^, ^25^, ^26^, ^27^

### 3.2 Prevalence

Patient‐reported cancer prevalence in jails or prisons was 0.4%–9.0% in three studies^6^, ^16^, ^24^ and in one study was comparable to that of the general public.^6^ In one survey study, White men reported higher cancer prevalence than Black men (1.3% vs. 0.4%).^24^ The most common cancers were cervical, lung, colorectal, non‐Hodgkin’s lymphoma, and hepatocellular carcinoma,^20^, ^21^, ^23^ and these cancers were more common in incarcerated than non‐incarcerated patient populations in the two studies that compared findings to SEER cancer prevalence.^21^, ^23^ One study found that women in jails and prison were at higher risk of cancer due to high rates of gender‐specific cancers,^22^ especially cervical cancer for which prevalence was high.^20^, ^22^, ^23^

### 3.3 Incidence

Zlotorzynska et al. is the only study to provide standardized cancer incidence ratios, but this registry‐based, 20‐year follow‐up of a cohort of individuals incarcerated in 1991 does not differentiate cancers diagnosed during or after incarceration.^12^ Nonetheless, it found that cancer incidence for HIV‐negative patients was slightly lower (standardized incidence ratio 0.9, 95% CI 0.8–0.9) than the general population in Georgia. The 4% of the cohort with HIV infection had higher cancer incidence than the general population (standardized incidence ratio 2.0, 95% CI 1.5–2.6). Consequently, the true cancer incidence for the total incarcerated population (which was not reported) may be slightly higher than the estimate for HIV‐negative patients and roughly equivalent to that of the general population. This study found that 36%, 23%, and 32% of patients were diagnosed with localized, regional, and metastatic disease, respectively, but no comparison to the non‐incarcerated population was reported. The most common cancers were lung, prostate, colorectal, kidney, and non‐Hodgkin lymphoma, consistent with the two other studies that provided data on cancer types.^20^, ^23^

### 3.4 Mortality

Data from all state prisons found that cancer accounted for 16,277 deaths (27% of all deaths) in state prisons from 2000 to 2018, growing from 58 to 92 per 100,000 incarcerated individuals over that period,^7^ but only 698 deaths (4% of all deaths) in local jails from 2000 to 2016.^17^ Cancer deaths in all state prisons were more common in White than Black and Hispanic patients (154 vs. 89 vs. 38 per 100,000 incarcerated individuals),^7^ findings echoed in data from Texas.^26^ Notably, these estimates are not adjusted for age, and do not include cancer deaths in federal prisons, which have a prison population approximately 1/10 of the combined state prison populations.

Among individuals incarcerated in Georgia prisons in 1991, Zlotorzynska et al. found that this cohort had higher cancer mortality (standardized mortality ratio (SMR) 1.2, 95% CI 1.1–1.3) than the general population in Georgia over the following 20 years.^12^ However, only 22% of these cancer deaths occurred during incarceration, while the remainder occurred after release.^10^ In North Carolina, White incarcerated men had higher age‐adjusted SMR for all cancers (1.5, 95% CI 1.3–1.9) compared to non‐incarcerated White men, while Black incarcerated men had lower mortality for all cancers (0.7, 95% CI 0.6–0.9) compared to non‐incarcerated Black men.^27^ In this study, 24% of incarcerated patient deaths were attributed to cancer versus 28% for the general population, but this finding was not standardized or statistically tested.

Finally, a study of patients with cancer incarcerated in Texas prisons found that these patients had a median overall survival of 21 months compared with 54 months for age, gender, and race‐matched SEER patients, though this analysis did not match on stage.^23^

### 3.5 Formerly incarcerated patients

Seven studies, including three studies with mixed populations of incarcerated and formerly incarcerated patients, reported data on formerly incarcerated patients: one study reported data on prevalence,^19^ one study reported data on incidence,^12^ and six studies evaluated cancer mortality in this population.^10^, ^12^, ^13^, ^18^, ^28^, ^29^

### 3.6 Prevalence

In a national survey of the general population, individuals who indicated criminal justice involvement in the prior year reported higher rates of lung (4.6% vs. 2.4%), cervical (13.8% vs. 9.1%), and alcohol‐related cancers (3.5% vs. 1.7%) compared to individuals without recent criminal justice involvement, but not for colon, breast, prostate, and smoking‐related cancers.^19^

### 3.7 Incidence

The cancer incidence estimates of individuals incarcerated in Georgia prisons discussed above included individuals who were currently and formerly incarcerated at the time of cancer diagnosis.^12^ A companion study that looked at causes of death over the subsequent 20 years for this cohort found that 78% of cancer deaths occurred outside of prison,^10^ providing indirect evidence that, for individuals with a history of incarceration, cancer diagnoses are likely more common after release into the community. No studies provide precise estimates on cancer estimates solely for formerly incarcerated patients.

### 3.8 Mortality

Cancer was the second leading cause of death among formerly incarcerated patients in Georgia at 15% of all deaths.^10^ In Washington State, cancer was the third leading cause of death after release from prison at 71 per 100,000 person‐years despite a median follow‐up of only 2 years, with released patients have a SMR of 1.9 compared to age, sex‐, and race‐matched US general population.^28^ A study of a subset of the same patients found that formerly incarcerated patients had higher cancer mortality than incarcerated patients in Washington state prisons (68 vs. 42 per 100,000 person‐years).^13^

In North Carolina, individuals formerly incarcerated in prisons had higher cancer mortality than the general population (SMR 3.9, 95% CI 3.3–4.6),^18^ with a separate study showing that formerly incarcerated White individuals in the state had higher cancer mortality (SMR 1.27, 95% CI 1.20–1.34) but formerly incarcerated Black individuals had lower cancer mortality compared to the general population (SMR 0.74, 95% CI 0.70–0.78).^29^

## 4 DISCUSSION

This scoping review found 20 studies evaluating cancer prevalence, incidence, and mortality in US jails and prisons. There are numerous gaps in the literature, but these studies demonstrate that cancer is a leading and increasing cause of death for patients incarcerated in prison and for formerly incarcerated patients, and these patient populations are likely at higher risk of cancer mortality than the general population. Cancer mortality in jails is low, likely due to the short period of incarceration and younger age distribution. Overall cancer incidence and prevalence may be relatively similar for incarcerated patients compared to the general population but elevated for specific diseases like cervical cancer, but the data are very limited and less clear. Overall, methodologically robust comparisons in cancer incidence and mortality between incarcerated, formerly incarcerated, and general populations are scarce.

Despite limitations of the included studies, there are four important points that stand out. First, many of the most common cancers in patients who have experienced incarceration––lung, cervical, colorectal, and hepatocellular cancers––are preventable or have effective screening. Thus, incarceration or release from prison may present a public health opportunity to provide cancer control interventions that can reduce risk and cancer disparities by facilitating access to care. Indeed, as 95% of incarcerated individuals are ultimately released and 78% of cancer deaths in the Georgia cohort occurred after release from prison,^10^ the public health benefits of improved cancer prevention and screening for incarcerated and formerly incarcerated patients accrue primarily to the communities that otherwise bear the costs of cancer diagnosis and treatment.

Second, incarcerated patients may receive delayed or substandard cancer screening and treatment, contributing to worse cancer mortality. Compared to the general population, the Georgia cohort study showed lower cancer incidence but higher cancer mortality,^12^ and a Texas study showed higher mortality for incarcerated patients with cancer.^23^ Both studies have methodologic limitations (a mixed incarcerated/formerly incarcerated patient population and insufficient risk adjustment, respectively) that prevent drawing of strong conclusions, and further investigation into access to and quality of cancer care for incarcerated and formerly incarcerated patients is needed.

Third, the relationship of incarceration and cancer disparities may vary by race and by state. Incarcerated Black men have lower rates of cancer death than incarcerated White and Hispanic men, a reversal of disparities typically seen in the general population (though not all studies adjusted for age). Furthermore, the North Carolina studies showed that both incarcerated and formerly incarcerated White men had worse age‐adjusted cancer mortality than never‐incarcerated White men, while the reverse was true for Black men.^27^, ^29^ It is possible that cancer care in prison is of lower quality than care received by White men in the community but better quality than care received by Black men, particularly in states such as North Carolina that have large racial disparities in cancer mortality in the general population,^3^ but additional research in this area is needed.

Formerly incarcerated individuals appear to be at higher risk of cancer mortality than both incarcerated and never‐incarcerated patients, but this disparity may also vary by state.^13^, ^28^ While many factors may mediate this disparity, formerly incarcerated individuals are often uninsured, due in part to suspension or cancelation of Medicaid enrollment upon incarceration, barriers to re‐enrollment upon release, and strict eligibility criteria, particularly in states that have not expanded Medicaid.^30^, ^31^ As studies have demonstrated that improved access to Medicaid reduces cancer mortality and reduces racial disparities in time to treatment in the community,^32^, ^33^ research is needed to determine how state polices on Medicaid enrollment and post‐release healthcare transitions may influence disparities in cancer mortality for formerly incarcerated individuals compared to incarcerated and never‐incarcerated individuals.

Fourth, possible incarceration‐related disparities in cancer remain invisible as much data are simply unavailable. Data comparing age‐adjusted cancer incidence for the general population to that of incarcerated and, separately, formerly incarcerated patients are absent. Similarly, there are no comparisons of stage of presentation or cancer care quality between incarcerated, formerly incarcerated, and general populations to assess whether these patient populations have delays in diagnosis or inferior care that may contribute to cancer disparities. An ongoing study will link state cancer registry and incarceration data to address some of these questions in Connecticut.^34^ As many of the studies in this review came from just a few states, similar studies are needed with a diverse array of states to provide a comprehensive view on the relationship between incarceration and cancer disparities. Such studies are painstaking to assemble given the fragmented nature of the US criminal justice system and appropriate safeguards for research involving incarcerated individuals that nonetheless increase barriers to studying cancer in this population. When layered upon incarcerated patients’ exclusion from Medicare and commercial payer databases, these barriers currently render possible disparities mostly invisible and additional research is necessary to help prioritize disease‐specific interventions for improved cancer control. A cancer registry variable that indicates incarceration status at the time of diagnosis would increase transparency and enable states to evaluate the care quality for which they are paying. Finally, there were only four studies that included jails, likely because data are especially fragmented among the more than 3000 county jails in the United States. The comprehensive jail mortality data indicates that cancer mortality is uncommon, but the transitory nature of jail populations may mean that the consequences of potentially inferior cancer care in jails occur after release.

This review has several limitations. Of studies outside of PubMed, the review only included studies from the Bureau of Justice Statistics; though the Bureau of Justice Statistics compiles data reported from the states, individual states may publish additional statistics on cancer in their incarcerated populations. Only 25% of the included studies reported data from within the past 10 years; US prison populations have declined slightly over that time and changes to insurance and Medicaid expansion during that time may change the relationship between incarceration and cancer disparities. However, the US prison population older than 55 continues to grow. Most studies focused on prisons in single states and there was considerable heterogeneity in study design; particularly given the potential importance of state demographics and healthcare policies, these study characteristics limit the ability to draw robust conclusions from individual study findings. Finally, accurate data on cancer diagnoses and outcomes are only a starting point for identifying and ameliorating disparities, and data on access to cancer prevention, screening, and quality of care for incarcerated patients are even harder to find and sorely needed.

## 5 CONCLUSION

There is a growing spotlight on the US criminal justice system and its long‐lasting effects on health for incarcerated individuals. While the inequities of the criminal justice system itself must be addressed, the current reality is that the US prison population is aging and a substantial proportion of individuals, particularly Black men, spend at least some time in jail or prison. This scoping review highlights that cancer mortality is common among incarcerated and formerly incarcerated patients and may be higher than the general population. A more nuanced understanding of the relationship between incarceration and disparities in cancer incidence and mortality is critical to inform efforts to remediate cancer disparities for patients who have been incarcerated and the communities in which they reside.

## CONFLICT OF INTEREST

The authors have no conflict of interest relevant to this work to declare.

## Supporting information

Table S1
